# A double‐blind randomized controlled trial of personalized upper‐alpha neurofeedback in children with ADHD

**DOI:** 10.1111/jcpp.70188

**Published:** 2026-06-21

**Authors:** Yuliang Wang, Ka Yi Hui, Siu Fung Lu, Qing Chuan Hannah Lui, Lawrence Ma, Suhail Theparambil Asharaf, Davis Lak, David Coghill, Patrick Ip, Tatia Mei Chun Lee, Winnie Wan Yee Tso

**Affiliations:** ^1^ Department of Paediatrics and Adolescent Medicine University of Hong Kong Hong Kong China; ^2^ Department of Psychology University of Hong Kong Hong Kong China; ^3^ Murdoch Children's Research Institute Melbourne Victoria Australia; ^4^ The University of Melbourne Melbourne Victoria Australia; ^5^ The Duchess of Kent Children's Hospital Hong Kong China; ^6^ The Hong Kong Children's Hospital Hong Kong China

**Keywords:** ADHD, neurofeedback, alpha oscillation, randomized controlled trial

## Abstract

**Background:**

Neurofeedback (NF) is a nonpharmacological approach for ADHD, but standard protocols often overlook individual EEG variability, potentially limiting efficacy. We report the first double‐blind randomized controlled trial of a session‐adaptive individualized upper‐alpha NF protocol in children with ADHD, based on individualized 1/f + alpha model.

**Methods:**

Forty‐eight children (6–12 years) diagnosed with ADHD were randomized to individualized upper‐alpha NF (*n* = 26) or sham feedback (*n* = 22). Participants completed eight 1‐h sessions over four weeks. The primary outcome was parent‐rated ADHD symptoms on the SWAN scale, measured at baseline, post‐treatment, and follow‐up. Secondary outcomes included PedsQL, CBCL, SDQ, and neuropsychological tests. Intention‐to‐treat linear mixed‐effects models assessed Time × Condition interactions; within‐group changes were evaluated via paired *t*‐tests. EEG learning was defined as the change in each session's upper‐alpha/theta‐to‐lower‐alpha ratio.

**Results:**

Blinding was preserved (Bang's Index: caregivers = 0.13; staff = 0.21). In the NF group, 21/26 children (80.7%) achieved ‘learner’ status versus 36.4% in sham. Both groups showed significant reductions in ADHD symptoms post‐treatment (SWAN Total: NF, *d* = −0.879, *p* < .001; sham, *d* = −1.261, *p* < .001), with no between‐group difference (*d* = −0.062, *p* = .881). At six months, NF maintained improvements in ADHD symptoms (SWAN Total: *d* = −0.713, *p* = .006), without superiority over sham (*d* = 0.221, *p* = .633). Neuropsychological testing revealed NF‐specific gains in Purdue Pegboard non‐preferred hand (*d* = 1.093, FDR *q* = .042) and faster CPT‐3 reaction time (*d* = −1.210, FDR *q* = .025).

**Conclusions:**

Personalized upper‐alpha NF produced higher EEG learning and selective motor‐speed improvements but did not outperform sham in reducing parent‐rated ADHD symptoms. Eight sessions sufficed for robust EEG modulation, yet non‐specific training effects likely drove clinical gains, indicating NF alone may not be an adequate ADHD treatment.

## Introduction

Attention‐deficit/hyperactivity disorder (ADHD) is one of the most prevalent mental health disorders among children and has been extensively researched. It affects approximately 5%–8% of the children around the world (Ayano, Demelash, Gizachew, Tsegay, & Alati, 2023; Polanczyk, Willcutt, Salum, Kieling, & Rohde, 2014). ADHD often continues into adulthood and significantly impacts academic, social, and occupational functioning. Established treatments, such as medications and behavioral therapies, are effective at alleviating symptoms in the short term, while their long‐term effectiveness remains debated (Coghill, 2019; Jensen et al., 2007; Swanson, 2019). Concerns over the growth suppression associated with stimulant medications have prompted calls for integrating nonpharmacological interventions to reduce lifelong medication exposure (Charach, 2020).

One nonpharmacological intervention is electroencephalography (EEG) biofeedback or neurofeedback (NF). This approach is believed to be able to enhance brain plasticity through operant conditioning principle (Sitaram et al., 2017) and offer sustained clinical benefits. However, although most of the previous NF study demonstrated significant pre‐post training effect, evidence of its specific efficacy remains inconclusive (Cortese et al., 2016; Gosling et al., 2025; Westwood et al., 2025). This lack of effect can be attributed to several limitations of NF (Arns, Heinrich, & Strehl, 2014). One problem is the low learning rate observed in previous NF trials, where only a small proportion of participants successfully learned to regulate their brainwaves following neurofeedback training (Arnold et al., 2021; Schönenberg et al., 2017), and the training failed to produce significant changes in the targeted biomarkers, such as the theta‐to‐beta ratio, when comparing post‐treatment to pre‐treatment outcomes (Schönenberg et al., 2017).

Similar to other Brain–Computer Interface (BCI)‐based systems, the effectiveness of neurofeedback hinges on the ‘learning’ process, where the human brain adapts and acquires the ability to control the system (Lotte & Jeunet, 2015). Effective BCI control requires a dynamic collaboration between the brain (the subject) and the machine (the decoder), with both the brain and the decoding algorithm adapting to improve their communication. However, traditional standard neurofeedback protocols rely on fixed mechanisms and thresholds such as slow cortical potential (SCP), theta‐to‐beta ratio (TBR), or sensorimotor rhythm (SMR), to read ADHD‐related brain activity, and apply the same standards across all participants and sessions. This is an oversimplified approach that fails to account for the inter‐ and intra‐individual variability in human brain oscillations, ultimately impeding the participants' learning process.

Recently some trials have adapted elements of personalized NF training approach for ADHD (Hasslinger, Bölte, & Jonsson, 2022; Purper‐Ouakil et al., 2022). However, these have been limited to selecting the most suitable protocol for each participant based on their resting‐state EEG profile, and address only a subset of the actual inter‐individual variation. The issue of intra‐individual variability, the session‐to‐session fluctuations in qEEG dynamics (Kondacs & Szabó, 1999), has to date received little attention.

Posterior α‐oscillations (8–13 Hz) constitute the dominant rhythm in the human EEG and are closely associated with attentional allocation, inhibitory control, and regulation of cortical excitability (Bonnefond & Jensen, 2024; Jensen & Mazaheri, 2010; Klimesch, 2012). Rather than reflecting a passive ‘idling’ state, α activity is increasingly understood as an active gating mechanism that suppresses task‐irrelevant information and facilitates the allocation of neural resources during attention‐demanding tasks. This mechanism is particularly relevant to attention‐deficit/hyperactivity disorder (ADHD), in which distractibility, inefficient attentional allocation, and impaired inhibitory control are core clinical features. Importantly, ADHD‐related α abnormalities are unlikely to reflect a simple excess or deficit in a fixed frequency band; instead, pediatric EEG studies suggest impaired task‐related modulation of α activity, including attenuated α desynchronization during attentional control and working memory encoding, with broader meta‐analytic evidence indicating reduced event‐related α decreases in ADHD (Lenartowicz et al., 2016; Michelini, Salmastyan, Vera, & Lenartowicz, 2022). Within the α range, upper‐alpha activity has been more closely linked to task‐specific cognitive processing, attentional engagement, and memory‐related performance, whereas lower‐alpha activity is often considered more related to general arousal or non‐specific task demands (Klimesch, 2012; Zoefel, Huster, & Herrmann, 2011). This distinction provides a rationale for upper‐alpha neurofeedback as a plausible approach to training a more efficient attentional‐control state in ADHD. Consistent with this rationale, upper‐alpha neurofeedback has been associated with improved cognitive performance in healthy adults (Yeh, Hsueh, & Shaw, 2021; Zoefel et al., 2011), and preliminary open‐label evidence in children with ADHD reported reductions in parent‐rated ADHD symptoms and improvements in cognitive performance following individualized upper‐alpha up‐regulation (Escolano, Navarro‐Gil, Garcia‐Campayo, Congedo, & Minguez, 2014). However, direct evidence remains limited, and no double‐blind randomized sham‐controlled trial has rigorously tested whether upper‐alpha neurofeedback produces specific clinical benefits beyond non‐specific neurofeedback effects in children with ADHD.

In the present study, we developed a personalized upper‐alpha neurofeedback protocol designed to account for both inter‐individual and intra‐individual variability in alpha oscillations. Here, ‘personalized’ refers to individualized and session‐adaptive calibration of the upper‐alpha neurofeedback target according to each child's qEEG spectrum, rather than preselection or stratification based on baseline qEEG phenotypes. Using a double‐blind, sham‐controlled randomized trial, we aimed to determine whether this protocol could induce neurophysiological learning and produce specific clinical and neuropsychological benefits beyond non‐specific neurofeedback effects in children with ADHD.

## Methods

### Participants

Sixty children (Figure 1) meeting DSM‐5 criteria for ADHD, aged 6–12 years, were recruited through pediatric out‐patient clinic or from the community in Hong Kong. Children with autism, other co‐morbid psychiatric or neurological disorders, or an IQ below 70 were excluded (see Table S1 for complete inclusion and exclusion criteria). Fifty‐one participants were enrolled and entered the randomization phase (Figure 1A). Informed consent was obtained from parents or guardians, with assent from the participants. This trial is registered on ClinicalTrials.gov (NCT06142786).

**Figure 1:**
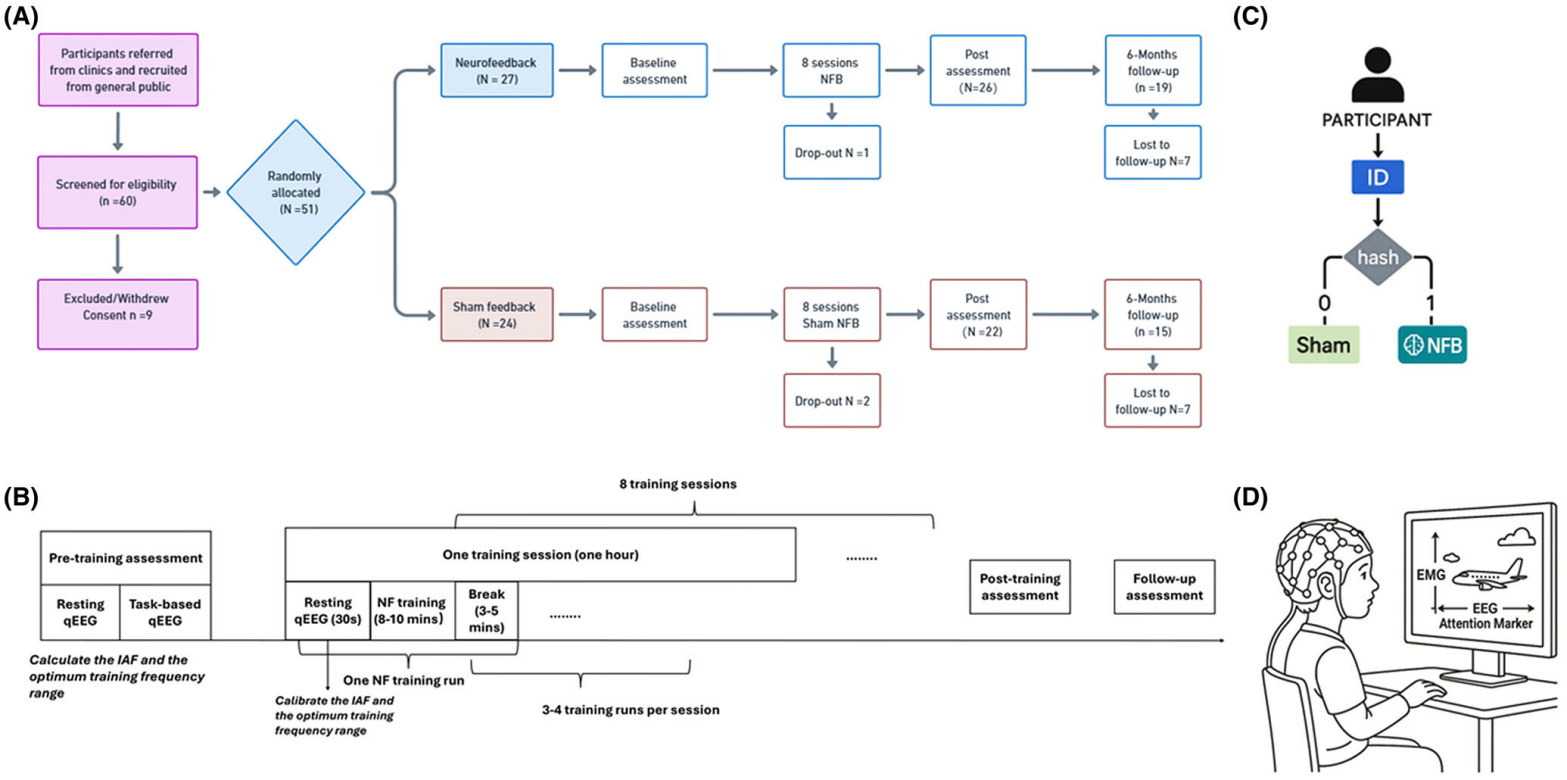
The study flow. (A) The recruitment flow chart of this randomized controlled trial. (B) The masking procedure of this randomized controlled trial. Each participant receives a unique de‐identified ID (for example, ‘NF001’) when first joining the study. The ID will be processed with a custom hashing function that produces a binary value (0 or 1) with equal probability. This determines whether they receive individualized alpha feedback or sham feedback. The neurofeedback software will present real or sham feedback automatically, without revealing allocation to either the investigators or the participants. Throughout the study, only the participant's de‐identified ID is visible to researchers, site staff, and participants. (C) The individualized Neurofeedback training protocol: (1) Pre‐training assessment: Resting‐state and task‐based quantitative electroencephalography (qEEG) recordings are acquired to derive individualized parameters, including the individual peak alpha frequency (IAF) and the optimal neurofeedback training frequency range. (2) Training phase: Each 60‐min session includes three sequential components: (i) a 30‐s resting‐state qEEG calibration to calibrate session‐specific baseline metrics, (ii) an 8‐ to 10‐min NF training run targeting the individualized frequency band, and (iii) a 3‐ to 5‐min rest interval. This sequence is repeated 3–4 times per session. Participants will complete a total of 8 sessions, administered over 4 weeks. (3) Post‐intervention evaluation: longitudinal follow‐up conducted 6 months post‐intervention. (D) Illustration of the neurofeedback paradigm. A child is seated at a desk wearing an electroencephalography (EEG) cap and viewing a computer screen displaying an airplane‐flight animation. Real‐time electromyography (EMG) activity from facial/neck muscles drives vertical movement of the plane while an EEG‐derived attention marker modulates its horizontal trajectory

### Masking and randomization procedure

Each participant receives a unique de‐identified ID (for example, ‘NF001’) when first joining the study. The ID was processed with a custom hashing function that produces a binary value (0 or 1) with equal probability. This determines whether they receive individualized alpha feedback or sham feedback. The neurofeedback software presented real or sham feedback automatically, without revealing allocation to either the investigators or the participants. Throughout the study, only the participant's de‐identified ID is visible to researchers, site staff, and participants, thus preserving blinding; see Figure 1C for the blinding diagram.

### Outcome measures

#### Primary outcome

Parent‐rated DSM‐based ADHD symptoms, measured through the Strengths and Weaknesses of ADHD‐symptoms and normal‐behaviors (SWAN). The Chinese SWAN is a validated instrument for the assessment of ADHD symptoms in Chinese children in Hong Kong (Lai et al., 2013). The SWAN questionnaire was originally devised based on 18 DSM‐IV ADHD symptoms. Scores range from −3 (far above normal) to +3 (far below normal), with 0 denoting average behavior.

#### Secondary outcomes

Parent‐report outcomes include (1) the Pediatric Quality of Life Inventory (PedsQL) (Varni, Seid, & Kurtin, 1999) a 23‐item self‐ and proxy‐report measure aimed at assessing health‐related quality of life across four subscales (Physical, Emotional, Social, and School Functioning) using a 5‐point Likert scale and linearly transformed to a 0–100 scale (higher = better) in scoring; (2) the ASEBA Child Behavior Checklist (CBCL) (Achenbach & Rescorla, 2001), a 113‐item survey designed to quantify emotional and behavioral problems across eight syndrome subscales using a 3‐point scale, higher scores indicate more problems; (3) the Strengths and Difficulties Questionnaire (SDQ) (Goodman, 1997), a brief scale targeting psychosocial adjustment via five subscales (Emotional Symptoms, Conduct Problems, Hyperactivity/Inattention, Peer Problems, and Prosocial Behavior) with responses on a 3‐point scale, higher scores indicate more problems.

Neuropsychological outcomes include WISC‐IV Processing Speed Index (Coding and Symbol Search) and Working Memory Index (Digit Span and Letter Number Sequencing) to assess processing speed and working memory (Wechsler, 2007) at baseline; the Purdue Pegboard Test (Tiffin & Asher, 1948) (Right, Left, Both Hands, Assembly) to evaluate fine motor dexterity by counting pegs placed or assemblies completed in timed trials; and the Conners' Continuous Performance Test‐3 (CPT‐3) (Conners, 2014) to measure sustained attention and response inhibition via presentations of target and non‐target stimuli.

### Neurofeedback intervention

The current neurofeedback study targeted up‐regulation of the personalized upper‐alpha band while down‐regulating the theta‐to‐lower‐alpha range. Individual alpha frequency (IAF) was determined by applying a 1/f + alpha model to the resting‐state EEG power spectrum using the FOOOF algorithm and identifying the alpha peak within the 7–13 Hz range (Donoghue et al., 2020b). The personalized upper‐alpha training band was defined as IAF to IAF + 2 Hz, following Zoefel et al. (2011), while the down‐training band was defined as IAF – 6 to IAF – 1 Hz. To account for intra‐individual changes in the EEG spectrum over time (Sidelinger, Zhang, Frohlich, & Daughters, 2023), IAF and the corresponding training bands were readjusted using the 1/f + alpha modeling approach before each neurofeedback training session. The full rationale and detailed protocol are provided in Appendix S1, Figures S1 and S2.

During neurofeedback, each child viewed an airplane‐flight animation (Figure 1D). Real‐time electromyographic (EMG) activity controlled the plane's vertical movement, while an EEG‐derived attention index – the ratio of power in the upper‐alpha band to power in the theta–lower‐alpha band – modulated its horizontal trajectory. The plane advanced only when this ratio increased, with forward speed proportional to the ratio magnitude. The initial reward threshold was determined based on the resting‐state training target ratio. After crossing the screen, the plane reset to a new starting position, indicating entry into the next level, and the reward threshold was readjusted based on the participant's performance in the previous level. In the sham‐feedback condition, all parameters were identical except that horizontal control was driven by prerecorded EEG from another child, randomly selected from an EEG neurofeedback database. Children in both groups completed eight real/sham neurofeedback sessions over four weeks, with two sessions per week. Each session lasted approximately one hour but was divided into 3–4 training runs of approximately 8–10 min each, separated by short breaks of approximately 3–5 min. Therefore, the total time actively engaged in the neurofeedback task was approximately 30–40 min per session. Site staff provided child‐friendly encouragement and reminders to maintain engagement, and parents supported children's cooperation and motivation when needed. The training scheme is illustrated in Figure 1B.

### EEG data analysis

#### Offline EEG analysis

EEG was acquired with a 32‐channel mBrainTrain system (10–20 system) at 500 Hz, band‐pass filtered 1–40 Hz. Data were re‐referenced to the common average; ocular and muscle artifacts were identified and removed by independent component analysis (ICA). IAF was estimated from eyes‐closed resting data via the FOOOF toolbox (Donoghue, Haller, et al., 2020b). QEEG spectra were computed with Welch's method (4 s Hamming windows, 50% overlap).

#### Online EEG processing

Real‐time EEG was streamed from Pz at 500 Hz, band‐pass filtered 1–40 Hz. Artifact suppression used the single trial PCA‐based artifact removal (SPA) algorithm (Ouyang, Dien, & Lorenz, 2022) on each 4 s sliding window. SPA‐cleaned data were segmented into 4 s epochs with 50% overlap (i.e. sliding windows advanced every 2 s), each epoch tapered with a Hamming window, and power spectral density was estimated via Welch's method. The real‐time visual feedback was provided based on the upper‐alpha/ theta‐to‐lower‐alpha ratio as noted in the previous section, update every 2 s. See Appendix S1 for detailed EEG processing methods.

### Statistical analysis

All analyses were performed in R (v4.4) using the lmerTest and broom.mixed packages. The intention‐to‐treat (ITT) includes all randomized participants with any postbaseline evaluation. For each outcome, we fitted a linear mixed‐effects model. with Time (Baseline, Post, Follow‐up), Condition (NF vs. Sham), and their interaction as fixed effects and a random intercept for each participant. Models were estimated by restricted maximum likelihood, and denominator degrees of freedom for fixed effects were approximated via the Satterthwaite method. Paired *t*‐tests were used to quantify within‐group changes (Baseline‐Post, Baseline‐Follow‐up), and between‐group effects were taken from the Time × Condition interaction. Missing data—both from drop‐outs and missed visits—were handled under full‐information maximum likelihood. To control the false discovery rate in our subscale analyses, *p* values were adjusted family‐wise using the Benjamini–Hochberg procedure. Sensitivity analysis was performed among ADHD children who never initiated medication (NF: *n* = 23; Sham: *n* = 18).

## Results

### Participant demographics

The study was conducted from Jan 2024 to March 2025 (Table 1). Out of the 60 children recruited, 9 were excluded or withdrew consent, leaving 51 participants for randomization. These participants were assigned to receive either individualized upper‐alpha neurofeedback (*n* = 27) or sham feedback (*n* = 24). All participants underwent a baseline assessment and then completed eight intervention sessions. One participant in the neurofeedback arm and two in the sham arm discontinued the intervention prior to post treatment assessment. Consequently, 26 neurofeedback and 22 sham participants completed the post‐treatment evaluation. At 6‐month follow‐up with questionnaire, outcome data were available for 19 participants in the neurofeedback group and 15 in the sham group; 7 participants in each group were lost to follow‐up, see Figure 1A. Table 1 presents demographic and baseline characteristics, showing no significant differences between groups in age, sex distribution, WISC working memory & processing speed index, or baseline symptom severity.

**Table 1:** Demographic characteristics of the participants

	Sham (*N* = 22)	NF (*n* = 26)	*p* Value^a^
	Mean/count (*SD*)	Mean/count (*SD*)	
Age	7.48 (1.82)	7.59 (1.93)	.840
Gender			
Male	17	20	
Female	5	6	.977
ADHD symptoms			
SWAN inattention	1.17 (0.47)	1.00 (0.52)	.252
SWAN hyperactivity	0.93 (0.43)	0.77 (0.55)	.263
SWAN Total	1.05 (0.32)	0.89 (0.48)	.168
ADHD medication			
Stimulant	4 (18.18%)	5 (19.23%)	.926
WISC			
Working memory index	92.64 (16.65)	91.27 (17.16)	.781
WISC			
Processing speed index	98.86 (20.61)	100.81 (19.07)	.738
Mother's education level			
Primary school	1 (4.55%)	2 (7.69%)	
Lower secondary school	3 (13.64%)	3 (11.54%)	
High school	6 (27.27%)	6 (23.08%)	
Diploma	5 (22.73%)	6 (23.08%)	
College degree	7 (31.82%)	9 (34.62%)	.987
Father's education level			
Primary school	0 (0.00%)	1 (3.85%)	
Lower secondary school	8 (36.36%)	9 (34.62%)	
High school	5 (22.73%)	3 (11.54%)	
Diploma	4 (18.18%)	4 (15.38%)	
College degree	5 (22.73%)	9 (34.62%)	.665
Family monthly income (in HKD)			
$ 4,000 or below	1 (4.55%)	0 (0.00%)	
$ 4,000–$ 7,999	2 (9.09%)	1 (3.85%)	
$ 8,000–$ 11,999	0 (0.00%)	1 (3.85%)	
$ 12,000–$ 15,999	1 (4.55%)	3 (11.54%)	
$ 16,000–$ 19,999	2 (9.09%)	0 (0.00%)	
$ 20,000–$ 29,999	3 (13.64%)	5 (19.23%)	
$ 30,000–$ 39,999	2 (9.09%)	1 (3.85%)	
$ 40,000–$ 49,999	1 (4.55%)	5 (19.23%)	
$ 50,000–$ 79,999	3 (13.64%)	6 (23.08%)	
$ 80,000–$119,999	3 (13.64%)	2 (7.69%)	
$120,000 or above	4 (18.18%)	2 (7.69%)	.403

^a^ *T*‐tests were used to compare continuous outcomes while chi‐square tests were used to compare categorical outcomes.

### Post‐hoc power analysis

We conducted a post‐hoc power analysis for the primary outcome, ADHD symptoms measured by the SWAN Total score. The between‐group effect size was defined as a difference‐in‐differences Cohen's *d*, representing the standardized difference in change from baseline between the NF and sham groups. Based on the available sample sizes, the minimum detectable between‐group effect sizes were *d* = 0.829 for T0–T1 (NF, *n* = 26; sham, *n* = 22) and *d* = 0.998 for T0–T2 (NF, *n* = 19; sham, *n* = 15).

### Blinding assessment

For caregivers, 27 of 48 guesses (56.3%) were correct, which did not differ from chance (χ^2^ = 0.75; *p* = .384). For site training staff, 29 of 48 guesses (60.4%) were correct (χ^2^ = 2.08; *p* = .150). Using Bang's Blinding Index (Bang, Ni, & Davis, 2004) (BI = 2·*P*correct–1), the BI was 0.13 for caregivers and 0.21 for staff (overall BI = 0.17), values close to zero that indicate minimal unblinding and thus successful masking of group assignment.

### Parent‐rated outcomes (Table 2, Figure 2)

**Table 2:** Comparison of parent‐rated outcomes between groups at baseline, post‐treatment, and 6‐month follow‐up

Outcome	Comparison	Group	Change^a^	*SD*	Effect size^b^	95% CI of effect size	*p* value^c^
SWAN Inattention	Baseline to End of Treatment	NF	−0.517	0.578	−0.895	(−1.30, −0.49)	<.001
	Baseline to End of Treatment	Sham	−0.545	0.446	−1.224	(−1.67, −0.78)	<.001
	Baseline to End of Treatment	Between	0.028	0.369	0.077	(−0.73, 0.88)	.852
	Baseline to 6‐Month F/U	NF	−0.456	0.570	−0.800	(−1.28, −0.32)	.003
	Baseline to 6‐Month F/U	Sham	−0.474	0.540	−0.878	(−1.43, −0.32)	.004
	Baseline to 6‐Month F/U	Between	0.149	0.388	0.384	(−0.52, 1.29)	.408
SWAN Hyperactivity	Baseline to End of Treatment	NF	−0.359	0.476	−0.754	(−1.16, −0.35)	<.001
	Baseline to End of Treatment	Sham	−0.293	0.419	−0.699	(−1.14, −0.26)	.004
	Baseline to End of Treatment	Between	−0.066	0.319	−0.207	(−1.01, 0.60)	.616
	Baseline to 6‐Month F/U	NF	−0.287	0.689	−0.416	(−0.90, 0.07)	.087
	Baseline to 6‐Month F/U	Sham	−0.222	0.585	−0.380	(−0.93, 0.17)	.163
	Baseline to 6‐Month F/U	Between	0.006	0.459	0.013	(−0.87, 0.90)	.978
SWAN Total Symptom Score	Baseline to End of Treatment	NF	−0.438	0.498	−0.879	(−1.28, −0.47)	<.001
	Baseline to End of Treatment	Sham	−0.419	0.332	−1.261	(−1.70, −0.82)	<.001
	Baseline to End of Treatment	Between	−0.019	0.305	−0.062	(−0.86, 0.74)	.881
	Baseline to 6‐Month F/U	NF	−0.371	0.521	−0.713	(−1.20, −0.23)	.006
	Baseline to 6‐Month F/U	Sham	−0.348	0.341	−1.020	(−1.57, −0.47)	.001
	Baseline to 6‐Month F/U	Between	0.072	0.325	0.221	(−0.68, 1.12)	.633
SDQ Total Score	Baseline to End of Treatment	NF	−2.385	2.729	−0.874	(−1.28, −0.47)	<.001
	Baseline to End of Treatment	Sham	−2.182	2.039	−1.070	(−1.51, −0.63)	<.001
	Baseline to End of Treatment	Between	−0.203	1.724	−0.118	(−0.92, 0.69)	.775
	Baseline to 6‐Month F/U	NF	−1.158	3.610	−0.321	(−0.80, 0.16)	.179
	Baseline to 6‐Month F/U	Sham	−1.000	2.104	−0.475	(−1.03, 0.08)	.087
	Baseline to 6‐Month F/U	Between	−0.186	2.177	−0.086	(−1.02, 0.85)	.859
CBCL Total Score	Baseline to End of Treatment	NF	−6.038	12.206	−0.495	(−0.90, −0.09)	.018
	Baseline to End of Treatment	Sham	−5.045	5.602	−0.901	(−1.34, −0.46)	<.001
	Baseline to End of Treatment	Between	−0.993	6.903	−0.144	(−0.95, 0.66)	.727
	Baseline to 6‐Month F/U	NF	−3.947	9.395	−0.420	(−0.90, 0.06)	.084
	Baseline to 6‐Month F/U	Sham	−2.800	8.360	−0.335	(−0.89, 0.22)	.216
	Baseline to 6‐Month F/U	Between	0.053	6.799	0.008	(−0.91, 0.92)	.987
PedsQL Total Score	Baseline to End of Treatment	NF	2.957	7.428	0.398	(−0.01, 0.80)	.053
	Baseline to End of Treatment	Sham	1.341	7.646	0.175	(−0.27, 0.62)	.420
	Baseline to End of Treatment	Between	1.616	5.323	0.304	(−0.50, 1.11)	.462
	Baseline to 6‐Month F/U	NF	2.941	7.693	0.382	(−0.10, 0.86)	.113
	Baseline to 6‐Month F/U	Sham	0.817	3.116	0.262	(−0.29, 0.82)	.327
	Baseline to 6‐Month F/U	Between	1.642	4.484	0.366	(−0.57, 1.30)	.450

^a^ Estimated change based on linear mixed model.

^b^ Cohen's *d* value.

^c^ Within‐group *p* values were estimated via paired *t*‐test and between‐group *p* value estimated via linear mixed model.

**Figure 2:**
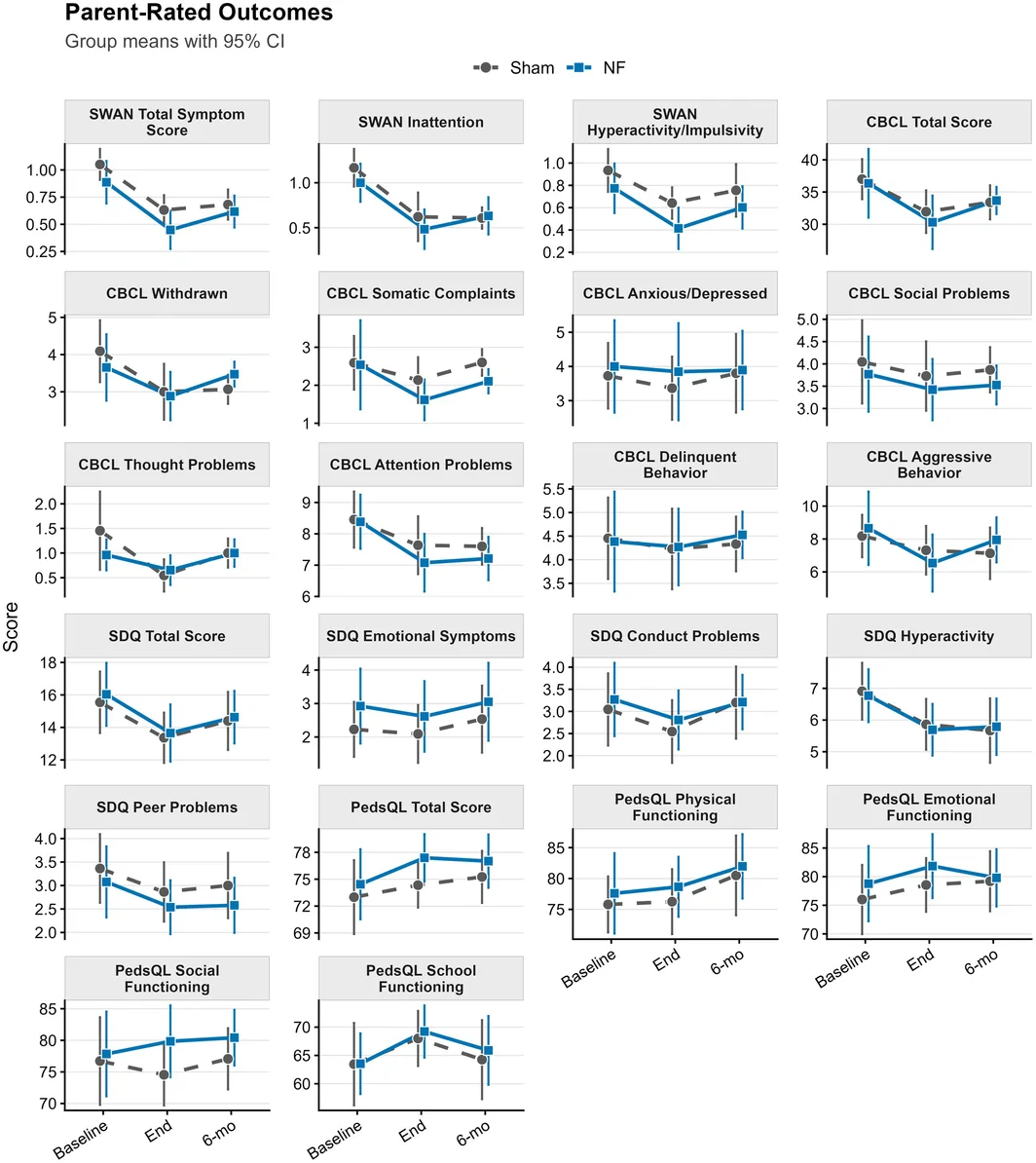
Parent‐rated outcomes at multiple time points. Error bars indicate ±95% CI

Both groups showed significant within‐group effects on core ADHD and behavioral measures by end of treatment (For NF group, SWAN Inattention, *d* = −0.895, *p* < .001; SWAN Hyperactivity/Impulsivity, *d* = −0.754, *p* < .001; SWAN Total, *d* = −0.879, *p* < .001; SDQ Total, *d* = −0.874, *p* < .001; CBCL Total, *d* = −0.495, *p* = .018), but none of these demonstrated significant between‐group differences (all between‐group *p* > .20). The within‐group improvements for NF group persisted at 6‐month follow‐up on SWAN Inattention (*d* = −0.800, *p* = .003) and SWAN Total symptoms (*d* = −0.713, *p* = .006), with neither change differing from sham (both between‐group *p* > .20). All other outcomes—SWAN Hyperactivity/Impulsivity (*d* = −0.416, *p* = .087), SDQ Total (*d* = −0.321, *p* = .179), CBCL Total (*d* = −0.420, *p* = .084), and PedsQL Total (*d* = 0.382, *p* = .113)—showed no significant change at 6‐month follow‐up compared with baseline. We also did not find between‐group differences among subscales of SDQ, CBCL, and PedsQL, see Figure 2 and Table S2 for subscale results. A sensitivity analysis among ADHD children who never initiated medication (NF: *n* = 23; Sham: *n* = 18) yielded results consistent with the main findings (see Table S3).

### Neuropsychological assessment outcomes (Table 3, Figure 3)

**Table 3:** Comparison of neuropsychological assessment outcomes between groups, at baseline, and post‐treatment

Outcome	Comparison	Group	Change^a^	*SD*	Effect size^b^	95% CI of effect size	*p* ^c^	FDR adjusted *p* ^d^
Pegboard Preferred Hand	Baseline to End of Treatment	Between	0.774	1.064	0.727	(−0.10, 1.55)	.082	.165
Pegboard Preferred Hand	Baseline to End of Treatment	Control	0.274	1.135	0.241	(−0.20, 0.68)	.270	.540
Pegboard Preferred Hand	Baseline to End of Treatment	NF	1.048	1.755	0.597	(0.19, 1.00)	.005	.**011**
Pegboard Non‐Preferred Hand	Baseline to End of Treatment	Between	1.299	1.189	1.093	(0.27, 1.92)	.011	.**042**
Pegboard Non‐Preferred Hand	Baseline to End of Treatment	Control	0.214	2.024	0.106	(−0.34, 0.55)	.626	.834
Pegboard Non‐Preferred Hand	Baseline to End of Treatment	NF	1.513	1.327	1.140	(0.74, 1.54)	<.001	**<.001**
Pegboard Both Hands	Baseline to End of Treatment	Between	0.375	1.470	0.255	(−0.57, 1.08)	.537	.685
Pegboard Both Hands	Baseline to End of Treatment	Control	0.394	1.613	0.244	(−0.20, 0.69)	.265	.540
Pegboard Both Hands	Baseline to End of Treatment	NF	0.769	2.400	0.320	(−0.08, 0.72)	.115	.153
Pegboard Assembly	Baseline to End of Treatment	Between	1.010	6.059	0.167	(−0.65, 0.98)	.685	.685
Pegboard Assembly	Baseline to End of Treatment	Control	0.159	6.553	0.024	(−0.42, 0.47)	.910	.910
Pegboard Assembly	Baseline to End of Treatment	NF	1.169	10.105	0.116	(−0.29, 0.52)	.561	.561
Conners Reaction Time *SD*	Baseline to End of Treatment	Between	−3.486	5.853	−0.596	(−1.42, 0.23)	.153	.255
Conners Reaction Time *SD*	Baseline to End of Treatment	Control	3.409	6.926	0.492	(0.05, 0.94)	.031	.156
Conners Reaction Time *SD*	Baseline to End of Treatment	NF	−0.077	9.260	−0.008	(−0.41, 0.40)	.967	.967
Conners Hit Reaction Time	Baseline to End of Treatment	Between	−4.388	3.627	−1.210	(−2.03, −0.39)	.005	.025
Conners Hit Reaction Time	Baseline to End of Treatment	Control	−0.227	1.510	−0.151	(−0.59, 0.29)	.488	.610
Conners Hit Reaction Time	Baseline to End of Treatment	NF	−4.615	6.818	−0.677	(−1.08, −0.27)	.002	.010
Conners Detectability	Baseline to End of Treatment	Between	−0.119	2.583	−0.046	(−0.87, 0.78)	.911	.911
Conners Detectability	Baseline to End of Treatment	Control	0.273	2.831	0.096	(−0.35, 0.54)	.656	.656
Conners Detectability	Baseline to End of Treatment	NF	0.154	4.221	0.036	(−0.37, 0.44)	.854	.967
Conners Commission Errors	Baseline to End of Treatment	Between	0.360	2.436	0.148	(−0.68, 0.97)	.720	.900
Conners Commission Errors	Baseline to End of Treatment	Control	0.409	2.062	0.198	(−0.25, 0.64)	.363	.605
Conners Commission Errors	Baseline to End of Treatment	NF	0.769	4.274	0.180	(−0.22, 0.58)	.368	.613
Conners Block Change	Baseline to End of Treatment	Between	−4.853	6.298	−0.771	(−1.60, 0.05)	.066	.166
Conners Block Change	Baseline to End of Treatment	Control	2.545	6.857	0.371	(−0.07, 0.81)	.096	.241
Conners Block Change	Baseline to End of Treatment	NF	−2.308	10.318	−0.224	(−0.63, 0.18)	.265	.613

^a^ Estimated change based on linear mixed model.

^b^ Cohen's *d* value.

^c^ Within‐group *p* values were estimated via paired *t*‐test and between‐group *p* values estimated via linear mixed model.

^d^ FDR‐adjusted *p* value using the Benjamini–Hochberg procedure to control the false discovery rate (FDR). Adjustment was applied separately within each outcome family (Pegboard, Conners) and comparison group (NF, Control, Between).

**Figure 3:**
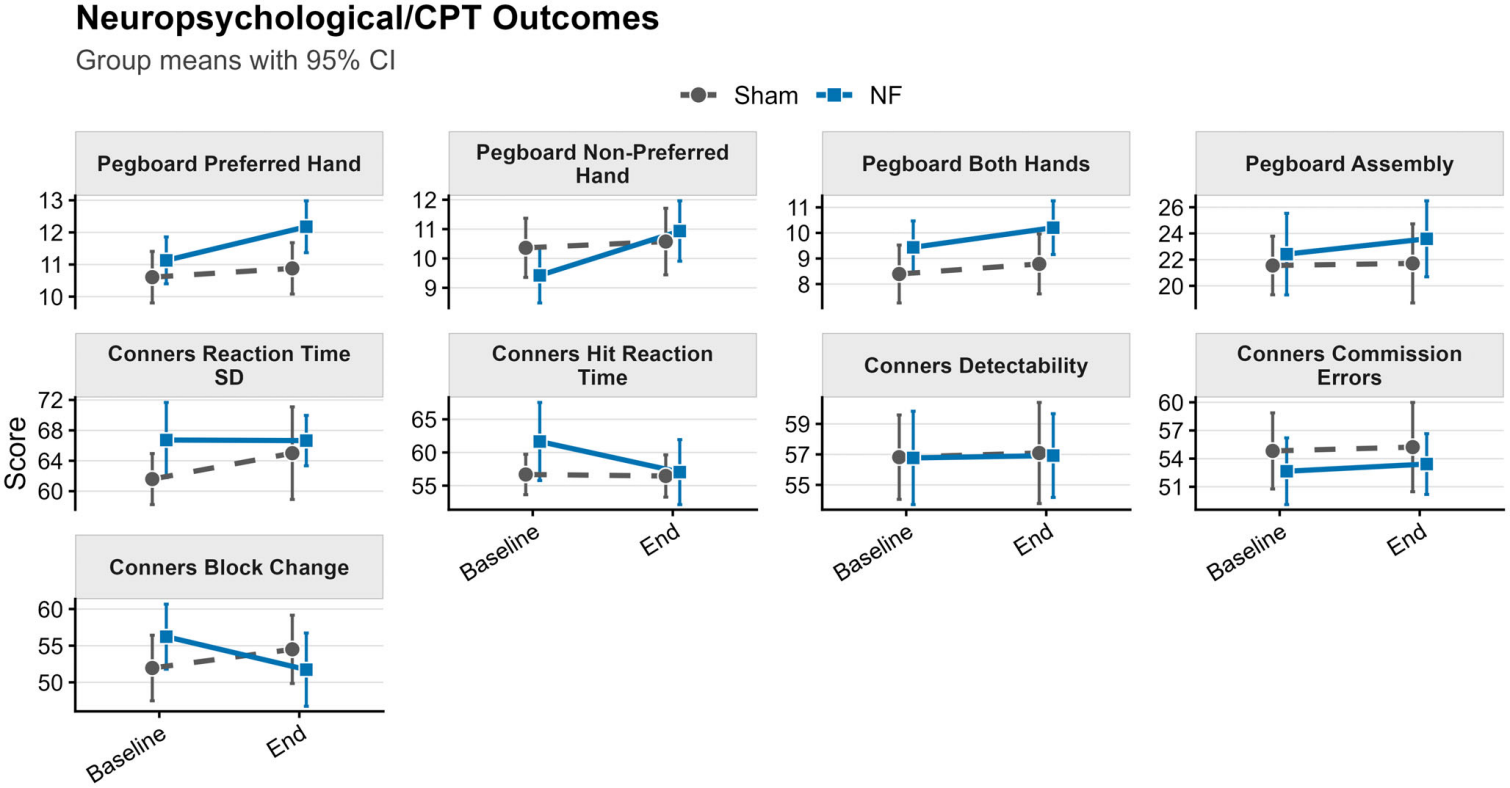
Neuropsychological assessment outcomes before and after the treatment. Error bars indicate ±95% CI

Within the NF group, Pegboard Preferred Hand (*d* = 0.597, *p* = .005, FDR *q* = .011), Pegboard Non‐Preferred Hand (*d* = 1.140, *p* < .001, FDR *q* < .001), and Conners Hit Reaction Time (*d* = −0.677, *p* = .002, FDR *q* = .010) showed significant change over time. Of these, Pegboard Non‐Preferred Hand (between‐group *p* = .011, FDR *q* = .042) and Conners Hit Reaction Time (between‐group *p* = .005, FDR *q* = .025) also demonstrated significant between‐group differences as compared to the sham group. No other neuropsychological measures demonstrated significant changes either within groups or between groups.

### EEG learning and neuromodulation effect

At intra‐session level, for each training run, participants in the NFB condition showed a 26.06% (95% CI ± 16.08%) increase in target training ratio, whereas those in the sham group increased by 6.06% (95% CI ± 14.08%), see Figure S3. At inter‐session level, the increase of EEG target training ratio was significantly higher than the sham group after 8 sessions of training, β = 23.31, *SE* = 5.55, *t* = 4.20, *p* < .001, *d* = 1.815, 95% CI [0.967, 2.663]. In the NF group, 21 of 26 participants (80.7%) were classified as ‘learners’ (i.e. higher targeted training ratio at the last training session compared to the first training session), whereas only 8 of 22 sham participants (36.4%) met this criterion, see Figure S4. See Appendix S2 for the complete EEG results.

### Adverse events

During the treatment period, no serious adverse events (SAEs) were reported in either group. General adverse events observed during training are summarized in Table S4.

## Discussion

To our knowledge, this is the first double‐blind randomized controlled trial (RCT) to assess a personalized upper‐alpha neurofeedback protocol in children with ADHD. Robust blinding outcome was achieved. Most previous neurofeedback studies have employed so‐called standard protocols (Arns et al., 2020), which generally do not incorporate adequate individualization procedures to accommodate intra‐ and inter‐individual EEG variability. In the present study, our individualized approach yielded a much higher learning rate (80%) compared with the standard theta/beta ratio (TBR (Arnold et al., 2021)) and slow cortical potential (SCP (Hasslinger et al., 2022)) protocols. Nevertheless, our findings largely replicate prior rigorously blinded trial results; although substantial within‐group changes were observed in the neurofeedback arm, there was no significant between‐group difference relative to sham treatment for core ADHD symptoms.

The non‐specific effects of sham neurofeedback can be attributed to several factors. First, parents' expectations may bias their subjective ratings of their children's ADHD symptoms. Second, sham treatment functions as a semi‐active intervention – it requires participants to remain seated for nearly an hour and to maintain covert attention on stimuli presented on the screen, both of which alone can enhance attentional control. Third, both neurofeedback and sham sessions typically include encouragement, praise for ‘trying hard’, and emotional/behavioral regulation tips (e.g. ‘Take a deep breath and focus on the screen’). These supportive interactions mirror aspects of behavioral therapy, fostering improvements in self‐esteem, motivation, and, ultimately, perceived symptom reduction.

Although the underlying mechanism of neurofeedback remains unclear, the ability to self‐regulate brainwaves is considered its key active component (Thibault, Lifshitz, & Raz, 2016). However, in previous rigorous trials of standard neurofeedback protocols, the learning rate in the NF group has only marginally beaten chance (Arnold et al., 2021; Hasslinger et al., 2022; Schönenberg et al., 2017). By contrast, our study demonstrated a superior learning rate within a brief training period. As recent neurofeedback literature indicates, personalized protocols typically outperform non‐personalized ones (Garcia Pimenta, Brown, Arns, & Enriquez‐Geppert, 2021; Hasslinger et al., 2022), and this is also true in broader BCI literature, where adaptive protocols generally outperform fixed protocols (Roc et al., 2021). Moreover, alpha oscillations have been shown to be easier to modulate both in neurofeedback training (Zoefel et al., 2011) and under varying cognitive demands (Bonnefond & Jensen, 2024), in contrast to the theta‐beta ratio, which correlates strongly with the trait‐liked 1/f aperiodic slope (Donoghue, Dominguez, & Voytek, 2020a) and may be difficult to alter over a short timeframe.

We observed significant within‐ and between‐group improvements on hit reaction time and single‐hand pegboard tasks following upper‐alpha neurofeedback. Although upper‐alpha NF in healthy young adults has been associated with improved attention and cognitive functions (Nan et al., 2024; Yeh et al., 2021; Zoefel et al., 2011), replication of such findings in pediatric samples is limited. Our findings of the increased motor processing speed align with a recent meta‐analysis of neurofeedback trials in children with ADHD, which identified processing speed as the only domain showing significant between‐group differences (Westwood et al., 2025). One possible explanation is that alpha NF primarily regulates cortical excitability (Jensen & Mazaheri, 2010; Lange, Oostenveld, & Fries, 2013), which may preferentially enhance functions that rely on optimal arousal, such as simple motor processing. However, because neuropsychological outcomes were secondary rather than primary outcomes in this trial, and multiple neuropsychological measures were examined, these findings should be interpreted with caution and require replication in future studies.

Our trial employed a shorter treatment duration of eight sessions. While this is shorter than many ADHD neurofeedback protocols, it aligns with other upper‐alpha neurofeedback protocols used for improving cognitive functions (typically eight to ten sessions (Yeh et al., 2021)). In standard neurofeedback protocols, most of the treatment effect had been achieved within the first half of the intervention period (Arnold et al., 2013, 2021; Schönenberg et al., 2017), calling into question whether longer courses are necessary to evaluate neurofeedback's efficacy. In our study, both the EEG modulation effect and the within‐group reduction on core ADHD symptoms—observed in both the neurofeedback and sham groups—were evident within this timeframe, indicating that an eight‐session protocol is sufficient to reproduce the large non‐specific effects reported previously. Moreover, because neurofeedback represents a considerable investment in terms of monetary expense, time, and opportunity cost, reducing the intervention to eight sessions can substantially cut costs and enhance cost‐effectiveness.

### Limitations

Several limitations of our study must be acknowledged. First, our sample size was modest. Although it was sufficient to detect significant within‐group changes, the study was powered only to detect relatively large between‐group effects (*d* > 0.83). Based on our post‐hoc power estimates, a future trial would require approximately 64 participants per group to detect a medium between‐group effect of Cohen's *d* = 0.50 with 80% power at α = .05. However, because the observed between‐group effects for the primary outcome were small (*d* < 0.30), the nonsignificant primary finding is unlikely to be explained solely by insufficient statistical power. Larger‐scale trials are nevertheless needed to detect smaller effects and to more definitively evaluate novel neurofeedback protocols. Second, we relied exclusively on parent‐rated outcome measures and did not include teacher assessments. While our rigorous blinding procedures helped mitigate rater bias, and previous studies have found minimal discrepancies between parent and teacher ratings in NF trials (Arnold et al., 2021), incorporating multi‐informant data would strengthen the generalizability of our findings. Third, we did not include a traditional neurofeedback comparator and therefore cannot determine whether personalized upper‐alpha neurofeedback is superior to standard protocols such as theta/beta ratio, sensorimotor rhythm, or slow cortical potential training. Our sham‐controlled design was intended to test whether the personalized protocol produced effects beyond non‐specific intervention factors, but not whether it offered incremental benefit over existing protocols. Fourth, we did not stratify participants by baseline qEEG phenotype. Although enrolling an unselected ADHD cohort improves real‐world generalizability, it may also increase heterogeneity and dilute treatment effects. Future adequately powered trials should compare personalized neurofeedback, traditional neurofeedback, and sham feedback, ideally with EEG‐profile‐based stratification, to determine whether individualized protocols improve learning efficiency and clinical outcomes in specific neurophysiological subgroups.

## Conclusion

While this personalized upper‐alpha neurofeedback protocol proved feasible and elicited robust EEG learning (80.7% learners) in children with ADHD and improved motor‐speed and hit reaction time, it did not produce greater reductions in parent‐rated core ADHD symptoms than sham feedback. This suggests that non‐specific effects may be driving much of the clinical benefit. Although adapting the target band to each child's peak alpha frequency enhanced neurophysiological modulation, translating these gains into broader symptom improvement remains challenging. Future research should further validate our findings on neurofeedback's effects on processing speed and explore whether these benefits can be extended to children with other developmental disabilities. Overall, the result does not support neurofeedback as a standalone treatment for ADHD.

## Ethical considerations

Informed consents/assent are obtained from all participants and their parents. The study protocol was approved by the Institutional Review Boards of Hospital Authority Central (Central IRB: PAED‐2023‐022) on 24 July 2023 and The University of Hong Kong/Hospital Authority Hong Kong West (HKU/HA HKW IRB: UW 23–296) on 10 Aug 2023.

## Trial registration

This trial is registered at ClinicalTrials.gov Protocol Registration and Results System (PRS). Ref No. NCT06142786. https://clinicaltrials.gov/study/NCT06142786. Record first released at 11/15/2023.

**Key points**

**What's known?**

- The learning efficiency and effectiveness of neurofeedback as an ADHD treatment remain under debate.

**What's new?**

- Using an 8‐week personalized upper‐alpha neurofeedback protocol, robust neuromodulation was achieved at both inter‐ and intra‐session levels in ADHD children.
- Both neurofeedback and sham training yielded significant but comparable reductions in core ADHD symptoms immediately post‐treatment, with effects persisting at the 6‐month follow‐up, indicating non‐specific effects.

**What's relevant?**

- Upper‐alpha neurofeedback produced significant improvements in motor processing speed in school‐aged ADHD children.

## Supporting information


**Table S1.** Inclusion, exclusion and drop‐out criteria.
**Table S2.** Subscale results of parent‐rated outcomes.
**Table S3.** Sensitivity analysis in medication‐Naïve ADHD participants (NF = 23; Sham = 18).
**Table S4.** Major adverse events reported.
**Appendix S1.** Methods – EEG processing method and neurofeedback protocol.
**Figure S1.** Top‐down view of our EEG electrode montage (international 10–20 system).
**Figure S2.** An illustration of the adaptive upper‐alpha neurofeedback protocol.
**Appendix S2.** EEG results.
**Figure S3.** Intra‐session change of EEG marker across conditions.
**Figure S4.** Inter‐session change of EEG marker across conditions.

## Data Availability

The analytical code and anonymized dataset are available from the corresponding author upon reasonable request.
