# Supplementary material for: A double‐blind randomized controlled trial of personalized upper‐alpha neurofeedback in children with ADHD
Source: J Child Psychol Psychiatry. 2026 Jun 21;67(12):1953–64. doi: 10.1111/jcpp.70188 (PMC13745793; doi:10.1111/jcpp.70188)
Supplement: Supplementary file 1 [file JCPP-67-1953-s001.docx]

**A Double-Blind Randomised Controlled Trial of Personalized Upper-Alpha Neurofeedback in Children with ADHD**

**Supporting Information**

**Table S1. Inclusion, Exclusion and Drop-Out Criteria.**

| **Category** | **No.** | **Criterion** |
| --- | --- | --- |
| **Inclusion** | 1 | ADHD diagnosis based on DSM-V criteria |
|  | 2 | Aged 6-12 |
|  | 3 | Wechsler Intelligence Test (WISC-IV IQ) ≥ 70 |
|  | 4 | Understand Chinese (Cantonese or Mandarin) |
|  | 5 | No ADHD medication taken, or on a stable regimen (≤ 20% change in dose/frequency) during the study period |
| **Exclusion** | 1 | Diagnosis of cerebral palsy or structural brain abnormalities on CT/MRI |
|  | 2 | Full-scale IQ < 70 |
|  | 3 | Comorbid conditions (epilepsy, ASD, affective disorders, other neurological disorders) |
|  | 4 | Participation in other non-pharmacological treatments or significant change of medication use (type/dosage) during the study period |
| **Dropout** | 1 | Voluntary withdrawal of consent by child or parent |
|  | 2 | Initiation of new medication or non-pharmacological treatment during the study period |
|  | 3 | Completion of fewer than half (4) of the training sessions |

**Table S2. Subscale results of Parent Rated Outcomes**

| Outcome | Comparison | Group | Change | SD | ES | CI_ES | p | p_adj | sig_FDR |
| --- | --- | --- | --- | --- | --- | --- | --- | --- | --- |
| SWAN Hyperactivity/Impulsivity | Baseline to Follow-Up | Between | 0.006 | 0.459 | 0.013 | (-0.87, 0.90) | 0.978 | 0.978 |  |
|  | Baseline to Follow-Up | Sham | -0.222 | 0.585 | -0.380 | (-0.93, 0.17) | 0.163 | 0.245 |  |
|  | Baseline to Follow-Up | NF | -0.287 | 0.689 | -0.416 | (-0.90, 0.07) | 0.087 | 0.156 |  |
| SWAN Inattention | Baseline to Follow-Up | Between | 0.149 | 0.388 | 0.384 | (-0.52, 1.29) | 0.408 | 0.525 |  |
|  | Baseline to Follow-Up | Sham | -0.474 | 0.540 | -0.878 | (-1.43, -0.32) | 0.004 | 0.013 | * |
|  | Baseline to Follow-Up | NF | -0.456 | 0.570 | -0.800 | (-1.28, -0.32) | 0.003 | 0.012 | * |
| SWAN Total Symptom Score | Baseline to Follow-Up | Between | 0.072 | 0.325 | 0.221 | (-0.68, 1.12) | 0.633 | 0.712 |  |
|  | Baseline to Follow-Up | Sham | -0.348 | 0.341 | -1.020 | (-1.57, -0.47) | 0.001 | 0.012 | * |
|  | Baseline to Follow-Up | NF | -0.371 | 0.521 | -0.713 | (-1.20, -0.23) | 0.006 | 0.014 | * |
| CBCL Aggressive Behavior | Baseline to Follow-Up | Between | 0.078 | 2.391 | 0.033 | (-0.90, 0.96) | 0.946 | 1.000 |  |
|  | Baseline to Follow-Up | Sham | -0.933 | 2.463 | -0.379 | (-0.93, 0.17) | 0.164 | 0.613 |  |
|  | Baseline to Follow-Up | NF | -1.000 | 3.815 | -0.262 | (-0.74, 0.22) | 0.268 | 0.613 |  |
| CBCL Anxious/Depressed | Baseline to Follow-Up | Between | -0.242 | 1.327 | -0.182 | (-1.12, 0.76) | 0.706 | 0.794 |  |
|  | Baseline to Follow-Up | Sham | 0.000 | 2.070 | 0.000 | (-0.55, 0.55) | 1.000 | 1.000 |  |
|  | Baseline to Follow-Up | NF | -0.263 | 1.695 | -0.155 | (-0.64, 0.33) | 0.507 | 0.762 |  |
| CBCL Attention Problems | Baseline to Follow-Up | Between | -0.312 | 1.224 | -0.255 | (-1.18, 0.67) | 0.593 | 0.762 |  |
|  | Baseline to Follow-Up | Sham | -0.800 | 1.521 | -0.526 | (-1.08, 0.03) | 0.061 | 0.565 |  |
|  | Baseline to Follow-Up | NF | -1.105 | 1.823 | -0.606 | (-1.09, -0.12) | 0.017 | 0.446 |  |
| CBCL Delinquent Behavior | Baseline to Follow-Up | Between | 0.392 | 1.269 | 0.309 | (-0.62, 1.24) | 0.520 | 0.762 |  |
|  | Baseline to Follow-Up | Sham | -0.267 | 1.387 | -0.192 | (-0.75, 0.36) | 0.469 | 0.762 |  |
|  | Baseline to Follow-Up | NF | 0.211 | 1.873 | 0.112 | (-0.37, 0.59) | 0.630 | 0.773 |  |
| CBCL Social Problems | Baseline to Follow-Up | Between | -0.506 | 1.226 | -0.412 | (-1.34, 0.51) | 0.390 | 0.702 |  |
|  | Baseline to Follow-Up | Sham | 0.533 | 1.807 | 0.295 | (-0.26, 0.85) | 0.272 | 0.613 |  |
|  | Baseline to Follow-Up | NF | -0.368 | 1.383 | -0.266 | (-0.75, 0.22) | 0.261 | 0.613 |  |
| CBCL Somatic Complaints | Baseline to Follow-Up | Between | -0.484 | 1.812 | -0.267 | (-1.16, 0.62) | 0.559 | 0.762 |  |
|  | Baseline to Follow-Up | Sham | 0.200 | 1.373 | 0.146 | (-0.41, 0.70) | 0.582 | 0.762 |  |
|  | Baseline to Follow-Up | NF | -0.789 | 3.190 | -0.247 | (-0.73, 0.23) | 0.295 | 0.613 |  |
| CBCL Thought Problems | Baseline to Follow-Up | Between | 0.493 | 1.088 | 0.453 | (-0.43, 1.34) | 0.318 | 0.614 |  |
|  | Baseline to Follow-Up | Sham | -0.600 | 2.098 | -0.286 | (-0.84, 0.27) | 0.287 | 0.613 |  |
|  | Baseline to Follow-Up | NF | -0.105 | 1.150 | -0.092 | (-0.57, 0.39) | 0.695 | 0.794 |  |
| CBCL Total Score | Baseline to Follow-Up | Between | 0.053 | 6.799 | 0.008 | (-0.91, 0.92) | 0.987 | 1.000 |  |
|  | Baseline to Follow-Up | Sham | -2.800 | 8.360 | -0.335 | (-0.89, 0.22) | 0.216 | 0.613 |  |
|  | Baseline to Follow-Up | NF | -3.947 | 9.395 | -0.420 | (-0.90, 0.06) | 0.084 | 0.565 |  |
| CBCL Withdrawn | Baseline to Follow-Up | Between | 0.656 | 1.263 | 0.519 | (-0.40, 1.44) | 0.275 | 0.613 |  |
|  | Baseline to Follow-Up | Sham | -0.933 | 1.831 | -0.510 | (-1.06, 0.04) | 0.068 | 0.565 |  |
|  | Baseline to Follow-Up | NF | -0.526 | 1.541 | -0.342 | (-0.82, 0.14) | 0.154 | 0.613 |  |
| PedsQL Emotional Functioning | Baseline to Follow-Up | Between | -2.996 | 9.401 | -0.319 | (-1.24, 0.60) | 0.503 | 0.636 |  |
|  | Baseline to Follow-Up | Sham | 5.867 | 11.501 | 0.510 | (-0.04, 1.06) | 0.068 | 0.424 |  |
|  | Baseline to Follow-Up | NF | 2.105 | 13.621 | 0.155 | (-0.33, 0.64) | 0.509 | 0.636 |  |
| PedsQL Physical Functioning | Baseline to Follow-Up | Between | -0.161 | 11.512 | -0.014 | (-0.91, 0.88) | 0.976 | 0.976 |  |
|  | Baseline to Follow-Up | Sham | 3.000 | 13.436 | 0.223 | (-0.33, 0.78) | 0.402 | 0.636 |  |
|  | Baseline to Follow-Up | NF | 3.553 | 16.673 | 0.213 | (-0.27, 0.70) | 0.365 | 0.636 |  |
| PedsQL School Functioning | Baseline to Follow-Up | Between | 4.680 | 7.462 | 0.627 | (-0.31, 1.56) | 0.198 | 0.595 |  |
|  | Baseline to Follow-Up | Sham | -1.067 | 8.746 | -0.122 | (-0.68, 0.43) | 0.644 | 0.743 |  |
|  | Baseline to Follow-Up | NF | 4.842 | 11.668 | 0.415 | (-0.07, 0.90) | 0.087 | 0.424 |  |
| PedsQL Social Functioning | Baseline to Follow-Up | Between | 3.648 | 10.785 | 0.338 | (-0.58, 1.25) | 0.474 | 0.636 |  |
|  | Baseline to Follow-Up | Sham | -4.533 | 10.239 | -0.443 | (-1.00, 0.11) | 0.108 | 0.424 |  |
|  | Baseline to Follow-Up | NF | 1.263 | 16.603 | 0.076 | (-0.41, 0.56) | 0.744 | 0.797 |  |
| PedsQL Total Score | Baseline to Follow-Up | Between | 1.642 | 4.484 | 0.366 | (-0.57, 1.30) | 0.450 | 0.636 |  |
|  | Baseline to Follow-Up | Sham | 0.817 | 3.116 | 0.262 | (-0.29, 0.82) | 0.327 | 0.636 |  |
|  | Baseline to Follow-Up | NF | 2.941 | 7.693 | 0.382 | (-0.10, 0.86) | 0.113 | 0.424 |  |
| SDQ Conduct Problems | Baseline to Follow-Up | Between | -0.136 | 1.412 | -0.096 | (-1.01, 0.81) | 0.837 | 1.000 |  |
|  | Baseline to Follow-Up | Sham | -0.067 | 1.624 | -0.041 | (-0.59, 0.51) | 0.876 | 1.000 |  |
|  | Baseline to Follow-Up | NF | -0.053 | 2.248 | -0.023 | (-0.51, 0.46) | 0.920 | 1.000 |  |
| SDQ Emotional Symptoms | Baseline to Follow-Up | Between | -0.000 | 1.285 | -0.000 | (-0.93, 0.93) | 1.000 | 1.000 |  |
|  | Baseline to Follow-Up | Sham | -0.133 | 1.959 | -0.068 | (-0.62, 0.49) | 0.796 | 1.000 |  |
|  | Baseline to Follow-Up | NF | -0.053 | 1.715 | -0.031 | (-0.51, 0.45) | 0.895 | 1.000 |  |
| SDQ Hyperactivity | Baseline to Follow-Up | Between | -0.245 | 0.766 | -0.320 | (-1.27, 0.63) | 0.513 | 1.000 |  |
|  | Baseline to Follow-Up | Sham | -0.667 | 1.175 | -0.567 | (-1.12, -0.01) | 0.045 | 0.340 |  |
|  | Baseline to Follow-Up | NF | -1.000 | 1.000 | -1.000 | (-1.48, -0.52) | <0.001 | 0.006 | * |
| SDQ Peer Problems | Baseline to Follow-Up | Between | -0.029 | 1.137 | -0.026 | (-0.95, 0.89) | 0.957 | 1.000 |  |
|  | Baseline to Follow-Up | Sham | -0.133 | 1.302 | -0.102 | (-0.66, 0.45) | 0.698 | 1.000 |  |
|  | Baseline to Follow-Up | NF | -0.053 | 1.747 | -0.030 | (-0.51, 0.45) | 0.897 | 1.000 |  |
| SDQ Total Score | Baseline to Follow-Up | Between | -0.186 | 2.177 | -0.086 | (-1.02, 0.85) | 0.859 | 1.000 |  |
|  | Baseline to Follow-Up | Sham | -1.000 | 2.104 | -0.475 | (-1.03, 0.08) | 0.087 | 0.435 |  |
|  | Baseline to Follow-Up | NF | -1.158 | 3.610 | -0.321 | (-0.80, 0.16) | 0.179 | 0.671 |  |
| SWAN Hyperactivity/Impulsivity | Baseline to End of Treatment | Between | -0.066 | 0.319 | -0.207 | (-1.01, 0.60) | 0.616 | 0.791 |  |
|  | Baseline to End of Treatment | Sham | -0.293 | 0.419 | -0.699 | (-1.14, -0.26) | 0.004 | 0.005 | * |
|  | Baseline to End of Treatment | NF | -0.359 | 0.476 | -0.754 | (-1.16, -0.35) | <0.001 | 0.001 | * |
| SWAN Inattention | Baseline to End of Treatment | Between | 0.028 | 0.369 | 0.077 | (-0.73, 0.88) | 0.852 | 0.881 |  |
|  | Baseline to End of Treatment | Sham | -0.545 | 0.446 | -1.224 | (-1.67, -0.78) | <0.001 | <0.001 | * |
|  | Baseline to End of Treatment | NF | -0.517 | 0.578 | -0.895 | (-1.30, -0.49) | <0.001 | <0.001 | * |
| SWAN Total Symptom Score | Baseline to End of Treatment | Between | -0.019 | 0.305 | -0.062 | (-0.86, 0.74) | 0.881 | 0.881 |  |
|  | Baseline to End of Treatment | Sham | -0.419 | 0.332 | -1.261 | (-1.70, -0.82) | <0.001 | <0.001 | * |
|  | Baseline to End of Treatment | NF | -0.438 | 0.498 | -0.879 | (-1.28, -0.47) | <0.001 | <0.001 | * |
| CBCL Aggressive Behavior | Baseline to End of Treatment | Between | -1.252 | 2.452 | -0.510 | (-1.31, 0.29) | 0.219 | 0.423 |  |
|  | Baseline to End of Treatment | Sham | -0.864 | 1.807 | -0.478 | (-0.92, -0.03) | 0.036 | 0.121 |  |
|  | Baseline to End of Treatment | NF | -2.115 | 4.403 | -0.480 | (-0.88, -0.08) | 0.022 | 0.083 |  |
| CBCL Anxious/Depressed | Baseline to End of Treatment | Between | 0.210 | 1.209 | 0.174 | (-0.63, 0.98) | 0.674 | 0.791 |  |
|  | Baseline to End of Treatment | Sham | -0.364 | 1.649 | -0.221 | (-0.66, 0.22) | 0.313 | 0.508 |  |
|  | Baseline to End of Treatment | NF | -0.154 | 1.759 | -0.087 | (-0.49, 0.32) | 0.660 | 0.791 |  |
| CBCL Attention Problems | Baseline to End of Treatment | Between | -0.490 | 1.192 | -0.411 | (-1.21, 0.39) | 0.322 | 0.508 |  |
|  | Baseline to End of Treatment | Sham | -0.818 | 1.181 | -0.693 | (-1.14, -0.25) | 0.004 | 0.034 | * |
|  | Baseline to End of Treatment | NF | -1.308 | 2.015 | -0.649 | (-1.05, -0.24) | 0.003 | 0.034 | * |
| CBCL Delinquent Behavior | Baseline to End of Treatment | Between | 0.112 | 1.135 | 0.099 | (-0.70, 0.90) | 0.811 | 0.842 |  |
|  | Baseline to End of Treatment | Sham | -0.227 | 1.378 | -0.165 | (-0.61, 0.28) | 0.448 | 0.636 |  |
|  | Baseline to End of Treatment | NF | -0.115 | 1.774 | -0.065 | (-0.47, 0.34) | 0.743 | 0.802 |  |
| CBCL Social Problems | Baseline to End of Treatment | Between | -0.028 | 1.183 | -0.024 | (-0.83, 0.78) | 0.954 | 0.954 |  |
|  | Baseline to End of Treatment | Sham | -0.318 | 1.524 | -0.209 | (-0.65, 0.23) | 0.339 | 0.508 |  |
|  | Baseline to End of Treatment | NF | -0.346 | 1.788 | -0.194 | (-0.60, 0.21) | 0.333 | 0.508 |  |
| CBCL Somatic Complaints | Baseline to End of Treatment | Between | -0.469 | 1.659 | -0.282 | (-1.09, 0.52) | 0.494 | 0.667 |  |
|  | Baseline to End of Treatment | Sham | -0.455 | 1.535 | -0.296 | (-0.74, 0.15) | 0.179 | 0.372 |  |
|  | Baseline to End of Treatment | NF | -0.923 | 2.855 | -0.323 | (-0.73, 0.08) | 0.112 | 0.283 |  |
| CBCL Thought Problems | Baseline to End of Treatment | Between | 0.601 | 0.881 | 0.683 | (-0.12, 1.49) | 0.102 | 0.283 |  |
|  | Baseline to End of Treatment | Sham | -0.909 | 1.444 | -0.629 | (-1.07, -0.19) | 0.008 | 0.051 |  |
|  | Baseline to End of Treatment | NF | -0.308 | 1.050 | -0.293 | (-0.70, 0.11) | 0.147 | 0.332 |  |
| CBCL Total Score | Baseline to End of Treatment | Between | -0.993 | 6.903 | -0.144 | (-0.95, 0.66) | 0.727 | 0.802 |  |
|  | Baseline to End of Treatment | Sham | -5.045 | 5.602 | -0.901 | (-1.34, -0.46) | <0.001 | 0.010 | * |
|  | Baseline to End of Treatment | NF | -6.038 | 12.206 | -0.495 | (-0.90, -0.09) | 0.018 | 0.083 |  |
| CBCL Withdrawn | Baseline to End of Treatment | Between | 0.322 | 1.555 | 0.207 | (-0.60, 1.01) | 0.616 | 0.791 |  |
|  | Baseline to End of Treatment | Sham | -1.091 | 1.925 | -0.567 | (-1.01, -0.12) | 0.015 | 0.079 |  |
|  | Baseline to End of Treatment | NF | -0.769 | 2.405 | -0.320 | (-0.72, 0.08) | 0.115 | 0.283 |  |
| PedsQL Emotional Functioning | Baseline to End of Treatment | Between | 0.531 | 6.430 | 0.083 | (-0.72, 0.89) | 0.841 | 0.854 |  |
|  | Baseline to End of Treatment | Sham | 2.545 | 12.003 | 0.212 | (-0.23, 0.66) | 0.331 | 0.710 |  |
|  | Baseline to End of Treatment | NF | 3.077 | 5.578 | 0.552 | (0.15, 0.96) | 0.009 | 0.137 |  |
| PedsQL Physical Functioning | Baseline to End of Treatment | Between | 0.603 | 7.947 | 0.076 | (-0.73, 0.88) | 0.854 | 0.854 |  |
|  | Baseline to End of Treatment | Sham | 0.455 | 8.952 | 0.051 | (-0.39, 0.49) | 0.814 | 0.854 |  |
|  | Baseline to End of Treatment | NF | 1.058 | 12.849 | 0.082 | (-0.32, 0.49) | 0.678 | 0.854 |  |
| PedsQL School Functioning | Baseline to End of Treatment | Between | 1.147 | 10.409 | 0.110 | (-0.69, 0.91) | 0.789 | 0.854 |  |
|  | Baseline to End of Treatment | Sham | 4.545 | 17.816 | 0.255 | (-0.19, 0.70) | 0.245 | 0.710 |  |
|  | Baseline to End of Treatment | NF | 5.692 | 11.492 | 0.495 | (0.09, 0.90) | 0.018 | 0.137 |  |
| PedsQL Social Functioning | Baseline to End of Treatment | Between | 4.182 | 9.041 | 0.463 | (-0.34, 1.27) | 0.265 | 0.710 |  |
|  | Baseline to End of Treatment | Sham | -2.182 | 10.084 | -0.216 | (-0.66, 0.23) | 0.322 | 0.710 |  |
|  | Baseline to End of Treatment | NF | 2.000 | 14.675 | 0.136 | (-0.27, 0.54) | 0.494 | 0.740 |  |
| PedsQL Total Score | Baseline to End of Treatment | Between | 1.616 | 5.323 | 0.304 | (-0.50, 1.11) | 0.462 | 0.740 |  |
|  | Baseline to End of Treatment | Sham | 1.341 | 7.646 | 0.175 | (-0.27, 0.62) | 0.420 | 0.740 |  |
|  | Baseline to End of Treatment | NF | 2.957 | 7.428 | 0.398 | (-0.01, 0.80) | 0.053 | 0.266 |  |
| SDQ Conduct Problems | Baseline to End of Treatment | Between | 0.038 | 1.113 | 0.035 | (-0.77, 0.84) | 0.933 | 0.933 |  |
|  | Baseline to End of Treatment | Sham | -0.500 | 1.566 | -0.319 | (-0.76, 0.12) | 0.149 | 0.280 |  |
|  | Baseline to End of Treatment | NF | -0.462 | 1.581 | -0.292 | (-0.70, 0.11) | 0.149 | 0.280 |  |
| SDQ Emotional Symptoms | Baseline to End of Treatment | Between | -0.171 | 1.143 | -0.150 | (-0.95, 0.65) | 0.716 | 0.933 |  |
|  | Baseline to End of Treatment | Sham | -0.136 | 1.885 | -0.072 | (-0.52, 0.37) | 0.738 | 0.933 |  |
|  | Baseline to End of Treatment | NF | -0.308 | 1.350 | -0.228 | (-0.63, 0.18) | 0.256 | 0.427 |  |
| SDQ Hyperactivity | Baseline to End of Treatment | Between | -0.031 | 0.772 | -0.041 | (-0.84, 0.76) | 0.921 | 0.933 |  |
|  | Baseline to End of Treatment | Sham | -1.045 | 1.046 | -1.000 | (-1.44, -0.56) | <0.001 | <0.001 | * |
|  | Baseline to End of Treatment | NF | -1.077 | 1.129 | -0.954 | (-1.36, -0.55) | <0.001 | <0.001 | * |
| SDQ Peer Problems | Baseline to End of Treatment | Between | -0.038 | 1.053 | -0.037 | (-0.84, 0.77) | 0.929 | 0.933 |  |
|  | Baseline to End of Treatment | Sham | -0.500 | 1.058 | -0.473 | (-0.92, -0.03) | 0.038 | 0.113 |  |
|  | Baseline to End of Treatment | NF | -0.538 | 1.772 | -0.304 | (-0.71, 0.10) | 0.134 | 0.280 |  |
| SDQ Total Score | Baseline to End of Treatment | Between | -0.203 | 1.724 | -0.118 | (-0.92, 0.69) | 0.775 | 0.933 |  |
|  | Baseline to End of Treatment | Sham | -2.182 | 2.039 | -1.070 | (-1.51, -0.63) | <0.001 | <0.001 | * |
|  | Baseline to End of Treatment | NF | -2.385 | 2.729 | -0.874 | (-1.28, -0.47) | <0.001 | <0.001 | * |

| Outcome | Comparison | Group | Change | SD | ES | CI_ES | p |
| --- | --- | --- | --- | --- | --- | --- | --- |
| SWAN Inattention | Baseline to End of Treatment | NF | -0.571 | 0.538 | -1.063 | (-1.52, -0.61) | <0.001 |
| SWAN Inattention | Baseline to End of Treatment | Sham | -0.593 | 0.429 | -1.380 | (-1.88, -0.88) | <0.001 |
| SWAN Inattention | Baseline to End of Treatment | Between | 0.021 | 0.347 | 0.061 | (-0.83, 0.95) | 0.894 |
| SWAN Inattention | Baseline to Follow-Up | NF | -0.556 | 0.556 | -0.999 | (-1.53, -0.47) | 0.001 |
| SWAN Inattention | Baseline to Follow-Up | Sham | -0.500 | 0.551 | -0.908 | (-1.49, -0.33) | 0.005 |
| SWAN Inattention | Baseline to Follow-Up | Between | 0.057 | 0.383 | 0.148 | (-0.82, 1.12) | 0.766 |
| SWAN Hyperactivity/Impulsivity | Baseline to End of Treatment | NF | -0.349 | 0.497 | -0.702 | (-1.16, -0.25) | 0.004 |
| SWAN Hyperactivity/Impulsivity | Baseline to End of Treatment | Sham | -0.364 | 0.347 | -1.050 | (-1.55, -0.55) | <0.001 |
| SWAN Hyperactivity/Impulsivity | Baseline to End of Treatment | Between | 0.015 | 0.307 | 0.049 | (-0.84, 0.94) | 0.915 |
| SWAN Hyperactivity/Impulsivity | Baseline to Follow-Up | NF | -0.229 | 0.724 | -0.317 | (-0.85, 0.22) | 0.225 |
| SWAN Hyperactivity/Impulsivity | Baseline to Follow-Up | Sham | -0.262 | 0.586 | -0.447 | (-1.02, 0.13) | 0.118 |
| SWAN Hyperactivity/Impulsivity | Baseline to Follow-Up | Between | 0.076 | 0.448 | 0.170 | (-0.78, 1.12) | 0.728 |
| SWAN Total Symptom Score | Baseline to End of Treatment | NF | -0.460 | 0.497 | -0.926 | (-1.38, -0.47) | <0.001 |
| SWAN Total Symptom Score | Baseline to End of Treatment | Sham | -0.478 | 0.243 | -1.966 | (-2.46, -1.47) | <0.001 |
| SWAN Total Symptom Score | Baseline to End of Treatment | Between | 0.018 | 0.283 | 0.064 | (-0.83, 0.95) | 0.889 |
| SWAN Total Symptom Score | Baseline to Follow-Up | NF | -0.392 | 0.564 | -0.695 | (-1.23, -0.16) | 0.014 |
| SWAN Total Symptom Score | Baseline to Follow-Up | Sham | -0.381 | 0.329 | -1.158 | (-1.74, -0.58) | <0.001 |
| SWAN Total Symptom Score | Baseline to Follow-Up | Between | 0.071 | 0.330 | 0.216 | (-0.75, 1.18) | 0.663 |

**Table S3. Sensitivity Analysis in Medication-Naïve ADHD Participants (NF = 23; Sham = 18)**

**Table S4. Major Adverse Events Reported**

| **Event Name** | **Time*** | **Neurofeedback Group** | | **Sham group** | |
| --- | --- | --- | --- | --- | --- |
|  |  | No. of participants | No. of  times | No. of participants | No. of  times |
| Fatigue | During the intervention sessions | 4 | 11 | 2 | 9 |
| Eye discomfort | During the intervention sessions | 0 | 0 | 1 | 2 |

*No major adverse events were reported after the intervention sessions.

**Appendix S1. Methods-- EEG Processing Method and Neurofeedback Protocol**

**Offline EEG Processing**

Continuous EEG was recorded at a sampling rate of 500 Hz using a mBrainTrain 32-channel cap (international 10–20 montage), with an online reference electrode placed midway between Cz and FCz.

**Preprocessing of EEG data**. Preprocessing was performed in MATLAB R2024a with EEGLAB (v2023). The continuous data were band-pass filtered between 1 and 40 Hz. High-pass filtering at 1 Hz or above is a common pre-processing practice and has been demonstrated to be a prerequisite for good ICA decomposition (1). Next, channels whose standard deviation exceeded four times the median absolute deviation of the channel-wise SD distribution were marked as bad and interpolated using EEGLAB’s spherical spline method. The data were then re-referenced to the common average and decomposed via extended Infomax independent component analysis (ICA). Independent components were automatically classified with the Multiple Artifact Rejection Algorithm (MARA), removing those with a probability ≥ 0.80, and the remaining components were inspected and excluded manually.

**Spectrum analysis**. The cleaned continuous EEG was segmented into 4 s epochs (2000 samples at 500 Hz) with 50 % overlap and tapered with a Hamming window. Each 4 s segment was then zero-padded to 2048 points before computing the FFT. This zero-padding yields a true spectral resolution of 1/4 s = 0.25 Hz, sampled at ≈0.244 Hz intervals across the spectrum.

**Individual Alpha Frequency (IAF).** Eyes‐closed resting EEG at Pz were analyzed with the FOOOF toolbox to estimate each subject’s individual alpha frequency (IAF). Power spectra were modeled as a combination of a 1/f aperiodic component and a single Gaussian peak (max_n_peaks = 1) in the 7–13 Hz range, with peak widths constrained between 0.5 and 3 Hz (peak_width_limits = [0.5, 3]). The center frequency of this Gaussian was taken as the IAF.

**Online EEG processing**

The online EEG signals will be collected via the Lab Streaming Layer (LSL), streaming from Pz at 500Hz and initially filtered using a 40 Hz low-pass filter and a 1 Hz high-pass filter. Artifactual signals will subsequently be removed using Signal Processing Algorithms (SPA). To ensure accurate feedback, EEG data for each feedback presentation will be recorded over a 4-second interval (comprising 2000 samples). Feedback will be provided every 2 seconds, incorporating both 2 seconds of new data and 2 seconds of previously collected data (each 4 s sliding window with 50 % overlap. Windows advanced every 2 s).

**Figure S1.** Top-down view of our EEG electrode montage (international 10–20 system). All recording sites are shown as black dots; the Pz electrode—our primary channel of interest, streaming source of the neurofeedback—is circled.

**Fine-tuning the upper alpha range based on task-based EEG data (pre-training).** Alpha‐band oscillations can exhibit both trait-like stability and state-dependent modulation under cognitive load. Samaha and Postle (2015) demonstrated that, for most individuals, the individual alpha frequency (IAF) measured during eyes-closed rest and simple visual tasks remains highly correlated and differs by less than 1 Hz, reflecting a stable neurophysiological signature (2). In contrast, Samuel et al. (2018) showed that IAF can shift by up to ~1–2 Hz during high-load encoding versus retention phases of a memory task, indicating dynamic tuning of cortical excitability (3). Because conventional neurofeedback protocols use a fixed window (IAF to IAF + 2 Hz) that may misalign reinforcement for responders whose peak alpha shifts under attention, an adaptive approach that tracks each subject’s functional IAF maximizes specificity and ensures that feedback targets the true upper-alpha rhythm driving attentional control. During the pre-training assessment period each participant completed a 3-minute eyes-closed resting EEG recording and a 10-minute covert‐attention task (visual oddball task) block, and IAF was defined as the spectral peak within 7-13 Hz for each block. If the task‐block IAF did not exceed the EC resting IAF by more than 1 Hz, the up-training feedback band was set to [IAF_rest, IAF_rest + 2 Hz], preserving the individual’s stable peak. If the task IAF exceeded EC resting IAF by > 1 Hz, the band was centered on the shifted peak ± 1 Hz ([IAF_task – 1 Hz, IAF_task + 1 Hz]), thereby capturing state-dependent acceleration.

**Figure S2**. An illustration of the adaptive upper-alpha neurofeedback protocol. If a participant’s task-block IAF did not exceed their eyes-closed resting IAF by more than 1 Hz, the up-training feedback band was set to [IAF_rest, IAF_rest + 2 Hz] (blue shading); if the task IAF exceeded the resting IAF by > 1 Hz, the band was centered on the shifted peak ± 1 Hz [IAF_task – 1 Hz, IAF_task + 1 Hz] (orange shading). In our cohort, only two participants in the NF group exhibited task IAF > resting IAF + 1 Hz and thus received the task-related alpha band for neurofeedback.

**Fine-tuning the upper alpha range (during training)** Before each training run, participants will complete a 30-second eyes-closed resting-state qEEG recording to recalibrate their IAF. If FOOOF identifies a clear peak within the target band, that new IAF will be used for the forthcoming training. If no valid peak is detected, the IAF determined during the pre-assessment will be retained.

**Targeted Training Ratio** Traditional upper-alpha neurofeedback—whether targeting absolute power in the [IAF-IAF+2] Hz window or using relative power ratios—can be confounded by non-specific EEG power surges (e.g. muscle or movement artifacts) and may inadvertently drive down non-target bands (e.g. beta) in a manner that conflicts with established theta/beta‐ratio protocols. To maximize specificity (for up-relative-alpha protocol), we instead combine up-training of upper-alpha ([IAF, IAF + 2 Hz]) with down-training of the theta–lower-alpha band ([IAF – 6 Hz, IAF – 1 Hz]).

**Appendix S2. EEG Results**

Post-hoc offline EEG analysis was conducted to examine the modulation effect of the neurofeedback training as compared to the sham effect. The feedback provided channel Pz was used in the data analysis, Figure S2. To avoid bias using dynamic IAF in the data analysis, fixed upper-alpha ratio power [10-12 Hz] over [4-9 Hz] was used in the following analysis.

**Intra-session changes**

For each participant, we first segmented every training run into 100 equally-sized time bins and computed the mean upper-alpha ratio within each bin. These run-level bin averages were then collapsed across all runs to yield a single 100-bin trace per participant. Finally, we computed the condition-level grand mean (and 95 % CI) at each of the 100 time points by averaging across participant in the same group. The neurofeedback (NFB) group exhibited a markedly greater increase in Upper Alpha Ratio over the course of each training run than the sham‐feedback control. On average, participants in the NFB condition showed a 26.06 % (95 % CI ± 16.08 %) increase from the first to the last time bin, whereas those in the sham group increased by only 6.06 % (95 % CI ± 14.08 %). Across these run‐level averages, 24 of 26 participants (92.3 %) in the NFB group showed a higher mean upper‐alpha ratio in the final 3 % than in the first 3 %, whereas only 13 of 22 participants (59.1 %) in the sham group exhibited this pattern, see figure S3.

**Figure S3**. **Intra-session change of EEG marker across conditions.** Grand‐mean percent‐change trajectories of Upper Alpha Ratio over 100 equally‐spaced time bins across each training run, comparing the neurofeedback (NFB) group (blue) with the sham‐feedback control group (green). Each training run last 8-10 minutes, and therefore each time bin in the plot represents 4-6 seconds of EEG data. Shaded bands represent ± 95 % CI around each group’s mean trace; solid lines show the group means.

**Inter-session changes**

Session-wise averaged upper-alpha ratio were obtained for the analysis of inter-session change of qEEG profile. The linear mixed‐effects analysis revealed a robust Group × Session interaction: from session 1 to session 8, participants in the neurofeedback (NFB) condition showed a markedly greater increase in percent change of the upper‐alpha ratio than those in the sham condition (β = 23.31, SE = 5.55, t = 4.20, p < .001). Standardizing this difference by the model’s residual variability yields a large effect size (Cohen’s d = 1.815, 95% CI [0.967, 2.663]). In the NF group, 21 of 26 participants (80.7%) were classified as “learners” (i.e., higher targeted training ratio at the last training session compared to the first training session), whereas only 8 of 22 sham participants (36.4%) met this criterion, see figure S4.

**Figure S4**. **Inter-session change of EEG marker across conditions.** Session‐wise percent‐change in Upper Alpha Ratio (relative to Session 1) for the neurofeedback (NFB) group and the sham‐feedback control group with shaded bands showing ±1.96 × SEM (95 % CI).

**Reference**

1.	Chaumon M, Bishop DV, Busch NA. A practical guide to the selection of independent components of the electroencephalogram for artifact correction. Journal of neuroscience methods. 2015;250:47–63.

2.	Samaha J, Postle BR. The speed of alpha-band oscillations predicts the temporal resolution of visual perception. Curr Biol. 2015;25(22):2985–90.

3.	Samuel IBH, Wang C, Hu Z, Ding M. The frequency of alpha oscillations: Task-dependent modulation and its functional significance. NeuroImage. 2018;183:897–906.
